# Excessive activation of ionotropic glutamate receptors induces apoptotic hair-cell death independent of afferent and efferent innervation

**DOI:** 10.1038/srep41102

**Published:** 2017-01-23

**Authors:** Lavinia Sheets

**Affiliations:** 1 Eaton-Peabody Laboratory Massachusetts Eye and Ear Boston, MA 02114, USA; 2 Department of Otolaryngology Harvard Medical School Boston, MA 02115, USA

## Abstract

Accumulation of excess glutamate plays a central role in eliciting the pathological events that follow intensely loud noise exposures and ischemia-reperfusion injury. Glutamate excitotoxicity has been characterized in cochlear nerve terminals, but much less is known about whether excess glutamate signaling also contributes to pathological changes in sensory hair cells. I therefore examined whether glutamate excitotoxicity damages hair cells in zebrafish larvae exposed to drugs that mimic excitotoxic trauma. Exposure to ionotropic glutamate receptor (iGluR) agonists, kainic acid (KA) or N-methyl-D-aspartate (NMDA), contributed to significant, progressive hair cell loss in zebrafish lateral-line organs. To examine whether hair-cell loss was a secondary effect of excitotoxic damage to innervating neurons, I exposed *neurog1a* morphants—fish whose hair-cell organs are devoid of afferent and efferent innervation—to KA or NMDA. Significant, dose-dependent hair-cell loss occurred in *neurog1a* morphants exposed to either agonist, and the loss was comparable to wild-type siblings. A survey of iGluR gene expression revealed AMPA-, Kainate-, and NMDA-type subunits are expressed in zebrafish hair cells. Finally, hair cells exposed to KA or NMDA appear to undergo apoptotic cell death. Cumulatively, these data reveal that excess glutamate signaling through iGluRs induces hair-cell death independent of damage to postsynaptic terminals.

Intense acoustic trauma or ischemic injury leads to accumulation of the excitatory neurotransmitter glutamate in the cochlea^1^,^2^,^3^,^4^. There is evidence that excess glutamate acts as a primary trigger for subsequent pathologies in noise-exposed cochleae, the most well-characterized effect being consequent swelling of postsynaptic afferent nerve terminals resulting from overactivation of AMPA-type GluRs^5^,^6^,^7^,^8^. By contrast, whether excess glutamate signaling damages hair cells—the sensory receptors of the auditory system—has not yet been fully examined. Presynaptic iGluRs that regulate neurotransmitter release have been observed in many areas of the central nervous system^9^, and several studies suggest that all three types of iGluR subunits—AMPA, Kainate, and NMDA—are expressed and presynaptically-localized in cochlear hair cells^10^,^11^,^12^,^13^,^14^. Yet whether excessive activation of iGluRs contributes to hair-cell damage has not been directly studied in a mammalian model system because it is difficult to discern whether hair-cell death in iGluR-agonist exposed cochleae is the result of damage to the hair cells themselves or collateral damage from injured postsynaptic nerve terminals^15^.

Zebrafish afford a useful model system to address whether glutamate toxicity damages sensory hair cells. Zebrafish hair cells are homologous to mammalian hair cells^16^,^17^,^18^,^19^,^20^, yet are optically accessible in whole larvae within the lateral line organ—a sensory organ used to detect the movement of water that contains clusters of superficially localized hair cells called neuromasts (NMs). Additionally, zebrafish hair cells are amenable to pharmacological manipulation, allowing for drug application and subsequent examination of hair-cell morphology and function. This is particularly advantageous for investigating hair-cell toxicity, as delivering drugs into the cochlea is challenging and can in and of itself damage sensory hair cells^21^.

I therefore determined whether glutamate excitotoxicity directly damages hair cells by examining lateral-line NMs of 5 to 6-day-old zebrafish larvae exposed to drugs that mimic glutamate-induced excitotoxic trauma. Exposure to the iGluR agonists kainic acid (KA) or N-methyl-D-aspartate (NMDA) contributed to significant, progressive hair-cell loss is both wild-type larvae and in *neurogenin1a* morphants—fish that have morphologically mature hair cells devoid of afferent and efferent innervation. Analysis of iGluR expression in isolated hair cells populations subsequently revealed that, similar to what has been previously reported in mammalian systems, AMPA-, Kainate and NMDA-type receptor subunits are expressed in zebrafish hair cells. KA and NMDA mediated hair-cell death is characterized by the formation of apoptotic bodies and activation of caspase-3. Cumulatively, these data indicate that excessive signaling through iGluRs induces apoptotic hair-cell death, and suggests cell death may be instigated through iGluRs on the hair cells themselves.

## Results

### KA exposure leads to swelling and bursting of postsynaptic afferent terminals

**Figure 1:**
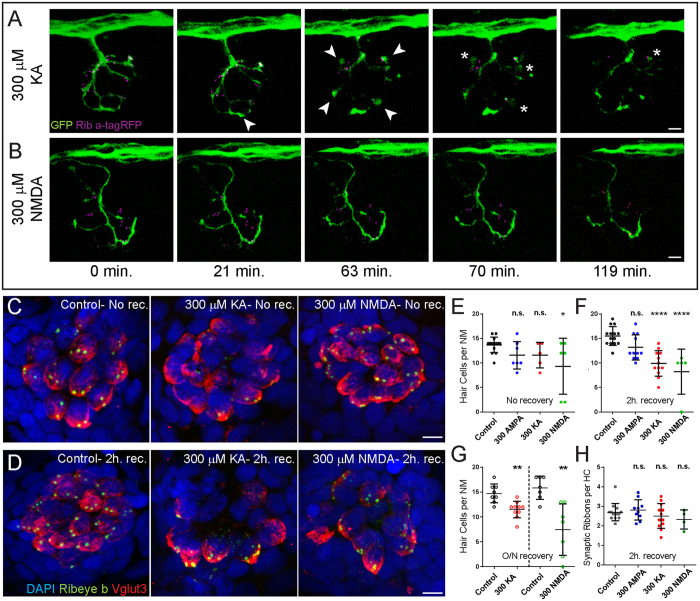
Exposure to KA or NMDA initiates hair-cell loss, but not synaptic-ribbon loss. (**A**,**B**) Live-imaging of KA (**A**) or NMDA (**B**) exposure: Representative frames from a movie taken over 2 hours with one frame per 3.5 minutes. Each frame is a maximum intensity projection (z-stack top-down image) of posterior lateral-line (LL) NM3 in a 6 dpf transgenic larva expressing Ribeye a-tagRFP in hair cells and GFP in afferent nerve fibers. Arrowheads in (**A**) indicate afferent-terminal swellings; asterisks indicate bursting afferent terminals. (**C**,**D**) Representative maximum intensity top-down (x-y) projections of imunolabeled Vglut3 (red) labeling the basolateral-end of hair cells and Ribeye b (green) labeling synaptic ribbons in NM3 of 6 dpf larvae. Larvae were exposed to DMSO carrier alone (Control) or iGluR agonists for 1 hour, then either immediately fixed ((**C**); No rec.) or rinsed and allowed to recover for 2 hours ((**D**); 2 h. rec.). Hair-cell death was confirmed by absence of DAPI-labeled nuclei. Scale bars: 3 μm (**E**–**H**) The number of intact hair cells per NM (**E**–**G**) or number of synaptic ribbons per hair cell (**H**) in 6 dpf (**E**,**F**,**H**) or 7 dpf (**G**) larvae exposed to DMSO alone or 300 μM iGluR agonist. Each circle represents NM3 in an individual larva. There was no significant loss of hair cells in NMs following exposure to AMPA at any time-point or the more excitotoxic AMPA/Kainate receptor agonist KA immediately following exposure (**E**). However, there were significantly fewer hair cells per NM in KA and NMDA-treated larvae compared to control 2 hours after drug exposure (F; ****p < 0.0001 defined by Dunnett’s multiple comparisons test) and after 20–22 h. recovery post-exposure (G, **p < 0.01 defined by unpaired t test). There was no significant difference in the number of synaptic ribbons per hair cell 2 h. after drug exposure (**H**), defined by Dunnett’s multiple comparisons test).

There is an abundance of evidence that cochlear nerve fibers are damaged by exposure to iGluR agonists: previous studies have reported excitotoxic damage to cochlear nerve fibers akin to that brought about by noise overexposure in cochleae briefly treated with the agonist α-amino-3-hydroxy-5-methyl-4-isoxazolepropionic acid (AMPA)^8^,^22^ or the more potent excitotoxic agonist KA^23^,^24^,^25^. To confirm whether zebrafish lateral-line afferent neurons are similarly sensitive to AMPA/KA GluR agonist-induced excitotoxic trauma, I exposed, to KA, live 6-day-old transgenic zebrafish larvae expressing GFP in their afferent neurons^26^ and mcherry at the hair-cell presynaptic ribbons while recording changes in their afferent terminal morphology using confocal time-lapse imaging. I observed profound swelling of lateral-line afferent terminals (Fig. 1A; white arrowheads) analogous to that observed in KA exposed mammalian cochleae^23^,^27^. In addition, I applied the iGluR agonist NMDA to these transgenic larvae and did not observe swelling of afferent terminals (Fig. 1B), which is also consistent with what had been previously observed in drug-perfused mammalian cochlea^5^. These observations support that iGluR agonists act on lateral-line afferent neurons in a similar manner as auditory dendrites.

### KA or NMDA exposure initiates NM hair-cell loss

Subsequently, I examined lateral-line NM hair-cell morphology in 5 to 6-day-old larvae that were exposed to AMPA, KA, or NMDA, then either immediately fixed or rinsed and allowed to recover either 2 hours or overnight prior to fixation. Unexpectedly, exposure to these high (300 μM) levels of either KA or NMDA contributed to significant, progressive hair cell loss (Fig. 1C–F) i.e. exposure to either KA or NMDA lead to noticeable hair-cell loss (Fig. 1E) that became more significant two hours after drug exposure (Fig. 1F). This result was surprising—the presence of functional iGluRs in sensory hair cells has been inferred by previous studies but not definitively established, so iGluR overactivation has not been considered as a cause of hair-cell death. Considering that KA exposure leads to swelling and bursting of afferent synaptic contacts (Fig. 1A), I also examined whether exposure to KA or NMDA lead to hair-cell synapse loss, but did not find any significant difference in the number of presynaptic ribbons per remaining NM hair cell in KA or NMDA exposed larvae (Fig. 1H). Cumulatively, these data show that activation of AMPA/Kainate or NMDA type receptors in zebrafish lateral line organs contributes to similar neural pathological changes as that observed in mammalian cochlea, and support that overactivation of iGluRs, possibly on the hair cells themselves, also contributes to noise-induced hair-cell damage and death.

### *neurogenin1* morphant NM hair cells appear morphologically mature and have functional mechanotransduction

**Figure 2:**
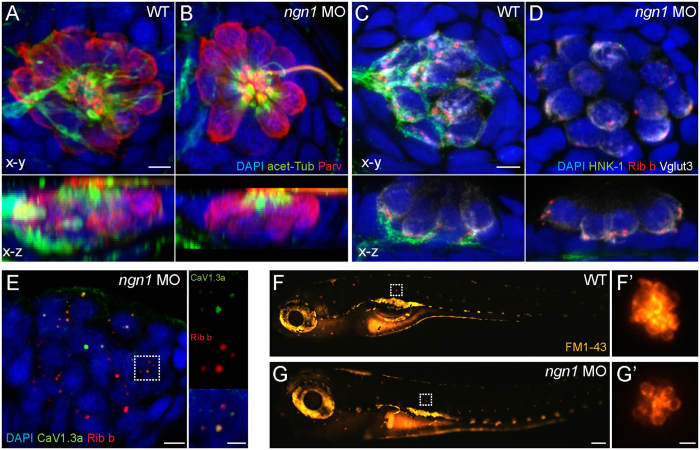
Characterization of *ngn1*1 morphant NM hair cells. (**A**,**B**) Representative max intensity top-down (x-y) and side-view (x-z) projections of imunolabeled Parvalbumin (red) labeling hair cells, acetylated-Tubulin (green) labeling neurons and the apical-region of hair cells, and DAPI labeling nuclei in posterior LL NM2 of WT (**A**) or *ngn1* MO (**B**) 5 dpf zebrafish larvae. Note the absence of immunolabeled green neurites beneath hair cells in the *ngn1* morphant NM (x-z) image. Scale bar: 3 μm (**C**,**D**) Representative immunoabeled (x-y) and (x-z) cross-sections of a WT (**A**) or *ngn1* MO(A’) anterior NM. Both synaptic ribbons (Ribeye; red) and synaptic vesicles (Vglut3; white) localize to the basolateral end of *ngn1* MO hair cells, despite the lack of afferent innervation (HNK-1; green). Scale bar: 3 μm (**E**) Max-intensity(x-y) projection of imunolabeled synaptic ribbons (red) and the presynaptic calcium channel Ca_V_1.3a (green) in NM3 of a 5 dpf *ngn1* morphant larva. Insets show colocalization of Ribeye and Ca_V_1.3, which is a hallmark of hair-cell maturation. Scale bars: 3 μm, 1 μm (insets) (**F**,**G**) Representative lateral views of 5 dpf WT (**F**) or *ngn1* morphant (**G**) larvae briefly exposed FM1-43. Insets show higher magnification top-down views of the NMs indicated by the white dashed squares. Labeling with FM1-43 indicates functional mechanotransduction machinery in *ngn1* morphant hair cells.

To exclude the possibility that damage to hair cells from exposure to KA or NMDA was an indirect result of overstimulating lateral-line nerve fibers, I examined *neurogenin1 (ngn1*) morphants: larvae whose hair-cell organs are devoid of afferent and efferent innervation^28^ (Fig. 2B). Zebrafish lacking *ngn1* are similar to mouse *ngn1* mutants in that their hair-cell organs lack innervation^29^. Ngn1-lacking zebrafish survive past the onset of lateral-line hair-cell function^30^ due to nutrition provided by the yolk sack.

Prior to exposing the larvae to drugs, I examined whether, in 5-day-old *ngn1* morphants, NM hair cells had the morphological characteristics of functionally mature hair cells. Immunolabled *ngn1* morphant hair cells showed several known hallmarks of morphological maturity: they had basally-localized presynaptic ribbons that localized with the synaptic-vesicle marker Vglut3 (Fig. 2D) as well as clusters of L-type calcium channels Cav1.3^31^ localized at the synaptic-ribbon (Fig. 2E). I also examined functional maturity by briefly exposing *ngn1* morphants to the vital dye FM1-43 at a concentration that has been previously shown to preferentially label relatively mature lateral-line NM hair cells with mechanotransduction activity^32^. Brief exposure to low concentrations of FM1-43 is a robust assay for functional hair-cell mechanotransduction that has been verified in both mammalian and zebrafish systems^33^,^34^,^35^. I observed that *ngn1* morphant NM hair cells take up FM1-43 (Fig. 2G), supporting that *ngn1*morphant zebrafish hair cells are morphologically and, at least in part, functionally mature despite lacking afferent and efferent innervation. It should be noted that hair-cell innervation is important for synaptic-ribbon maintenance in zebrafish^36^ and afferent innervation plays a significant and variable role in long-term hair-cell maintenance and viability in mice^37^. Nevertheless, previous studies have shown that short term hair-cell survival is possible in the absence of innervation^38^, indicating *ngn1* morphants as a useful tool in determining whether iGluR agonist exposure damages hair cells in the absence of innervation.

### KA or NMDA exposed *ngn1* morphants show NM hair-cell loss that is comparable to wild-type and dose dependent

**Figure 3:**
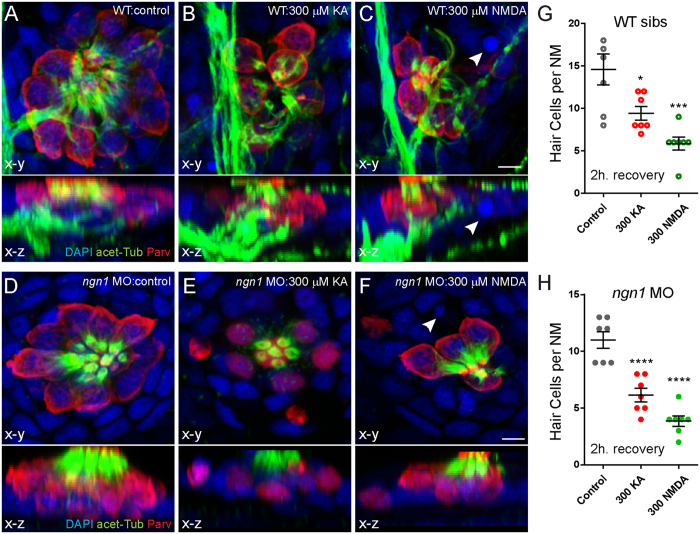
KA or NMDA exposure initiates hair-cell loss in *ngn1*1 morphants comparable to WT siblings. (**A**–**F**) Representative max intensity top-down (x-y) and side-view (x-z) projections of imunolabeled Parvalbumin (red), acetylated Tubulin (green), and DAPI (blue) in posterior LL NM7 of 5 dpf WT (**A**–**C**) or *ngn1* MO (**D**–**F**) larvae. Larvae were exposed to DMSO carrier alone (Control) or iGluR agonists for 1 hour, then rinsed and allowed to recover for 2 hours. Exposure to 300 μM KA (**B**,**E**) or 300 μM NMDA (**C**,**F**) leads to hair-cell loss in both WT and morphants. White arrowheads indicate pyknotic nuclei. Scale bar: 3 μm (**G**,**H**) The number of intact hair cells per NM in 5 dpf larvae exposed to DMSO alone, 300 μM KA, or 300 μM NMDA. Each circle represents NM2 or NM7 in an individual larva. There were significantly fewer HCs per NM in KA and NMDA-treated larvae compared to control 2 h. after exposure in both WT siblings (**G**) and *ngn1* (**H**) MO larvae (*p < 0.05; ***p < 0.001; ****p < 0.0001 defined by Dunnett’s multiple comparisons test).

**Figure 4:**
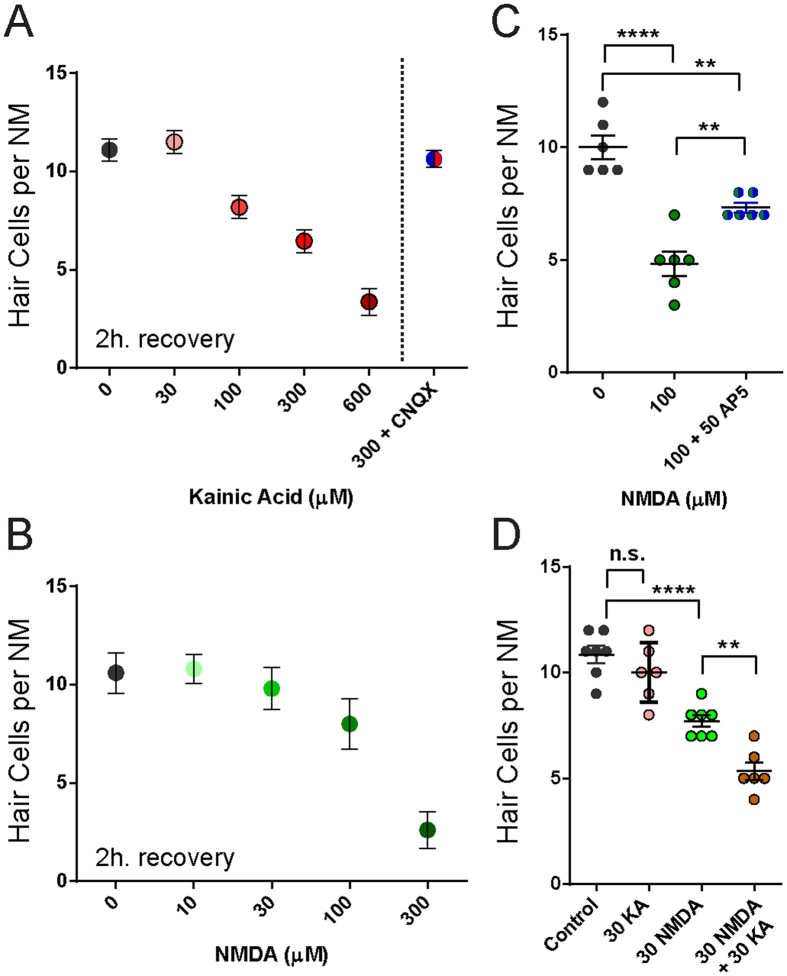
KA and NMDA induce hair-cell loss in a dose-dependent manner and have a synergistic effect in *ngn1* morphants. (**A**,**B**) Dose-dependent hair-cell loss in *neurog1* MO larvae exposed to KA (**A**) or NMDA (**B**). Each dot represents the mean hair cell loss per NM in 5–10 individual larvae; the bars represent the s.e.m. Co-exposure with the AMPA/KA receptor antagonist CNQX (300 μM) blocks KA-induced hair-cell loss (**A**). (**C**) NMDA-exposed NM hair cells are partially protected from NMDA mediated death when pre-exposed to the competitive NMDA receptor antagonist D-AP5. Each circle represents NM2 or NM7 in an individual larva. (**p < 0.01; ****p < 0.0001 defined by Dunnett’s multiple comparisons test). (**D**) The effect of combined KA and NMDA exposure on NM hair-cell death was greater than the sum of the effects of each drug alone. There were significantly fewer HCs per NM in larvae treated with a sub-lethal concentration of KA (**A**) and NMDA than NMDA alone. (**p < 0.01; ****p < 0.0001 defined by Dunnett’s multiple comparisons test).

Consequently, I exposed 5-day-old *ngn1* morphants and their WT siblings to KA or NMDA for 1 h., then allowed them to recover for 2 h. prior to fixation. Significant hair-cell loss occurred in *ngn1* morphants exposed to 300 μM KA or NMDA (Fig. 3D–H), and the loss was comparable to that observed in wild-type siblings (Fig. 3A–C,G). The actions of KA and NMDA are likely specific, as hair-cell loss in *ngn1* morphants occurred in a dose-dependent manner (Fig. 4A,B). Additionally, KA-induced hair-cell loss was blocked when morphants were co-exposed to the competitive AMPA/kainate receptor antagonist CNQX (Fig. 4A) and NMDA-induced hair-cell loss was partially blocked by NMDA receptor antagonist D-AP5 (Fig. 4C). The actions of KA and NMDA appeared to be synergistic; a non-lethal concentration of 30 μM KA combined with a minimally lethal concentration of 30 μM NMDA lead to significantly more hair-cell death than 30 μM NMDA alone (Fig. 4D; Control = 11 ± 0.4, 30 μM KA = 10 ± 0.6, 30 μM NMDA = 8 ± 0.3; 30 μM KA + 30 μM NMDA = 5 ± 0.4). To analyze the synergistic effect of KA and NMDA combined, I calculated the coefficient of drug interaction (CDI; see Methods)^39^. The CDI of KA + NMDA (30 μM) was 0.7; CDI < 1 indicates synergism. Cumulatively, these data suggest that excessive glutamate signaling mediates damage to sensory hair cells independent of damage to postsynaptic terminals, and implies the presence of functional iGluRs on hair cells.

### Zebrafish hair cells express AMPA-, KA-, and NMDA-type receptor subunits

**Figure 5:**
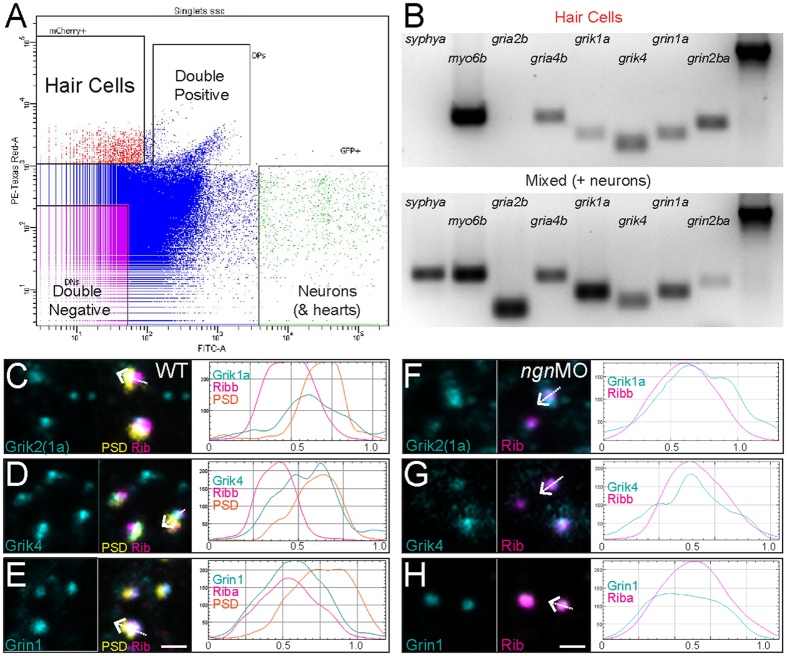
AMPA-, Kainate-, and NMDA-receptor subunits are expressed in zebrafish hair cells. (**A**) Fluorescence-activated cell sorting: fluorescent scatter was used to separate cell populations according to the fluorescent intensity of mcherry (hair cells) or GFP (neurons and hearts). (**B**) Gene expression analysis of isolated mcherry-expressing hair cells relative to the GFP-expressing mixed cell population. Gel images were cropped and inverted for display. The presence of *myo6b* expression was used as a positive control for hair cells and the absence of *syphya* expression was used as a negative control to ensure the sorted hair cell population was free of neurons. *β-actin* (far-right lane) was used as a loading control. Unedited gel images can be found in Supplementary Info. (**C**–**E**) Representative max-intensity images of immunolabeled wild-type hair-cell synapses: GluR subunits (cyan), synaptic ribbons labeled with Ribeye (magenta), and PSDs labeled with MAGUK (yellow). The antibody used to detect Grik1a was generated using a conserved region of the C-terminus of human Grik2 that has 92% percentage sequence conservation with zebrafish Grik1a (ClustalW). To the right of each image are corresponding profile plots showing fluorescent signal intensity (y-axis) in relation to distance (x-axis; μm). The white arrow in each merged image indicates the position and direction of the sampling line used to measure each corresponding profile plot. Note that immunolabel for and Kainate-receptor subunits Grik1a (**C**) and Grik4 (**D**) show multiple peak intensities that overlap with both Ribeye and MAGUK, while the NMDA-receptor subunit Gria1a (**E**) shows a single peak intensity that mostly overlaps with Ribeye. (**F**–**H**) Representative max-intensity images of immunolabeled *ngn1MO* hair-cell synapses. To the right of each image are corresponding profile plots. Note the immunolabel for Grik1a (**F**), Grik4 (**G**), and Grin1 (**H**) is present in the absence of postsynaptic densities and overlaps with synaptic ribbons. Scale bars: 1 μm.

Data from previous studies suggest that mammalian hair cells express AMPA-10,11, Kainate-12, and NMDA-type^14^,^40^ GluR subunits. To examine whether zebrafish hair cells express iGluR subunits, I performed gene expression analysis on hair cells isolated from 5-day-old stable transgenic larvae. Fluorescence-activated cell sorting (FACS) of lysed larval populations was used to separate mcherry containing hair cells and GFP containing neurons from other non-fluorescent cell types (Fig. 5A). To perform a survey of iGluR expression, cDNA templates were created from the sorted cells via RT-PCR, then used to generate small amplicons (~150–300 bp) of GluR transcripts of the AMPA, Kainate, and NMDA receptor classes as well as cell-type specific control genes. Using this strategy, I identified several iGluR subunits in zebrafish hair cells: AMPA-receptor subunit g*ria4b*, Kainate-receptor subunits *grik1a* and *grik4*, and NMDA-receptor subunits *grin1a* and *grin2ba* (Fig. 5B).

In addition, I detected immunolabeling of three identified iGluR subunits at hair-cell ribbon synapses. The commercial antibody used to detect zebrafish Grik1a was generated using a 49aa region of the C-terminus of human Grik2, which has 92% sequence conservation with zebrafish Grik1a aa 652–701 (ClustalW Method). I observed Grik1a (Fig. 5C), Grik4 (Fig. 5D), and Grin1a (Fig. 5E) immunolabeled puncta localized to hair-cell ribbon synapses. To determine the labeling distribution of iGluRs relative to both pre- and post-synaptic components, I compared iGluR labeling intensity plotted in relation to distance (profile plots) with that of synaptic ribbons and the afferent postsynaptic densities (PSDs). Figure 5(C–E) shows high-power images of representative ribbon synapses and their corresponding profile plots. Kainate-receptor subunits Grik1a (Fig. 5C) and Grik 4 (Fig. 5D) labeling showed multiple peak intensities that overlapped with both Ribeye and PSD peak intensities, suggesting these iGluR subunits localize to both pre- and postsynaptic regions. By contrast, NMDA-receptor subunit Grin1 showed a single peak intensity that overlapped with Ribeye labeling the presynapse (Fig. 5E). To determine whether GluR subunit labeling persisted in the absence of the postsynapse, I examined iGluR subunits labeling in *ngn1* morphant fish, and observed Grik1a, Grik 4, and Grin1a labeling localized with Ribeye (Fig. 5F–H). These results indicate iGluR subunits are expressed in zebrafish hair cells, and suggest that KA and NMDA damage to hair cells may be mediated by hair cell autoreceptors.

### KA or NMDA-induced hair-cell death is not accompanied by intracellular calcium ([Ca2+]_I_) dysregulation

**Figure 6:**
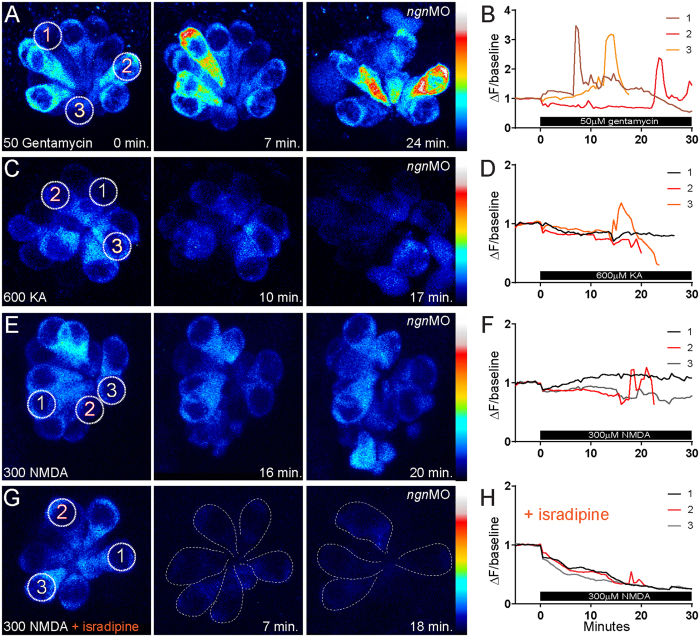
Cytoplasmic Ca^2+^ dynamics in hair cells treated with gentamycin vs. KA or NMDA. (**A**,**C**,**E**,**G**) Heat-mapped, time-lapse imaging of *ngn1* morphant NMs with hair cells stably expressing GCaMP3. Z-stack images were taken every 30 seconds for 30–50 minutes total. 50 μM gentamycin (**A**), 600 μM KA (**C**) or 300 μM NMDA (**E**,**G**) was applied 5 minutes into imaging; 10 μM isradipine (**G**) was applied prior to imaging and remained for the duration. Circles indicate the regions of interest (ROIs) where fluorescence changes in GCaMP3 were measured. Numbers correspond to cells measured for fluorescence intensity traces. Dashed outlines in (**H**) indicate hair cells; note the reduction in hair-cell number during NMDA exposure. (**B**,**D**,**F**,**H**) Transformed (ΔF/baseline) fluorescence intensity data. Three cells from each NM are depicted; the red and orange traces correspond to dying hair cells. Cells were chosen to highlight the difference in calcium transients in dying cells exposed to genatmycin (**B**) vs. iGluR agonists (**D**,**F**). Note the reduction in intracellular calcium in (**H**) does not provide protection from hair-cell death.

One mechanism of excitotoxic cell death is dysregulation of [Ca2+]_I_ homeostasis followed by the loss of mitochondrial membrane potential^41^. To examine whether [Ca2+]_I_ dysregulation occurs in hair cells exposed to KA or NMDA, I exposed *ngn1* morphant larvae stably expressing the genetically encoded calcium indicator GCaMP3 in hair cells^42^ to either KA or NMDA while recording changes in GCaMP3 fluorescence intensity using confocal time-lapse imaging. To verify I could visualize [Ca2+]_I_ dysregulation in *ngn1* morphant hair cells, I exposed them to 50 μM gentamycin, which has been previously shown in this transgenic line to induce a marked increase in cytosolic [Ca2+]_I_ prior to hair-cell death^42^. Likewise, I also observed sharp peaks in GCaMP3 fluorescence relative to baseline in gentamycin exposed hair cells prior to their death and extrusion from the NM (Fig. 6A,B). By contrast, I observed very little change in GCaMP3 fluorescence relative to baseline in dying hair cells exposed to either KA (Fig. 6C,D) or NMDA (Fig. 6E,F); dying hair cells exhibited small increases in GCaMP3 fluorescence prior to cell death (Fig. D (Cell 3); Fig. F (Cell 2)), but they were ~9 fold smaller than the calcium peaks observed in dying gentamycin-treated hair cells (Fig. 6B).

The majority (>90%) of total Ca^2+^ current in hair cells is carried by the L-type calcium channel Ca_V_1.3^18^,^31^,^43^,^44^. To further examine whether [Ca2+]_I_ dysregulation contributes to iGluR mediated hair-cell death, I inhibited extracellular calcium influx with the L-type calcium channel blocker isradipine at a concentration (10 μM) that has been previously shown to reduce calcium responses in zebrafish NM hair cells^45^. Consequently, I observed a dramatic reduction in GCaMP3 fluorescence in NM hair cells co-exposed to isradipine (Fig. G,H). Notably, blocking extracellular calcium influx did not provide a protective effect; the NM co-exposed to NMDA and isradipine lost hair cells (Fig.G; white dashed outline), which was never observed in NM hair cells exposed to isradipine alone^45^. Cumulatively, these data support that KA and NMDA do not bring about hair-cell death through dysregulation of [Ca2+]_I_ homeostasis and mitochondrial collapse.

### KA or NMDA exposed *ngn1*morphant NM undergo apoptotic cell death

**Figure 7:**
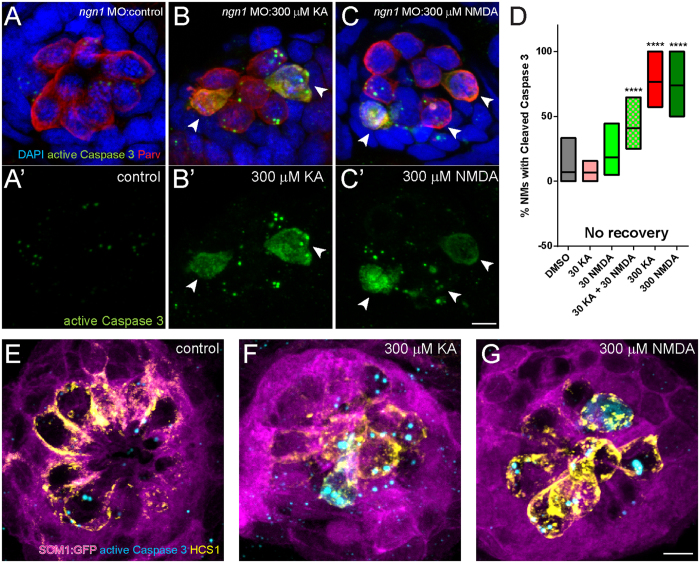
KA and NMDA exposed NMs contain hair cells with activated Caspase-3. (**A**–**C**): Representative max intensity top-down (x-y) images of NM7 in 5 dpf *ngn1* morphant larvae exposed to DMSO alone (**A**), 300 μM KA (**B**), or 300 μM NMDA (**C**), then immediately processed for histology. NMs exposed to iGluR agonists stained positively for activated Caspase-3, indicating the cells were undergoing apoptotic cell death. Scale bar: 3 μm (**D**) Floating bar graph showing the percentage of NM in individual larval posterior lateral line that stained positive for activated Caspase-3. Each bar represents the min. and max. values in 11–15 larvae; the lines represent the mean values. (****p < 0.0001 defined by Dunnett’s multiple comparisons test). (**E**–**G**) Representative max intensity images of NMs in transgenic 5 dpf larvae expressing GFP in lateral-line supporting cells (scm1:GFP) exposed to DMSO alone (**E**), 300 μM KA (**F**), or 300 μM NMDA (**G**), then then immediately processed for histology. Supporting cells (magenta) appear intact in drug-exposed larvae, and activated caspase-3 immunolabel (cyan) corresponds with the hair-cell marker HCS-1 (yellow). Scale bar: 3 μm.

Hair cells exposed to acoustic trauma have been shown to undergo apoptotic cell death, necrotic cell death, or a combination of the two^46^. To determine whether hair-cell death in iGluR-agonist exposed hair cells appears characteristically apoptotic or necrotic, I examined changes in hair-cell morphology in *ngn1* morphant larvae to either KA or NMDA. Exposure to either KA (Fig. 6C; Supplemental Fig. 1A) or NMDA (Fig. 6E; Supplemental Fig. 1B) led to the formation of blebs and what appeared to be apoptotic bodies accompanied by hair-cell extrusion from the NMs. I subsequently examined whether caspase-3—a downstream effector of apoptotic cell death—was activated in hair cells exposed to high concentrations (300 μM) of KA or NMDA, and found the majority of NMs contained a subset of hair cells with activated caspase-3 immediately following drug exposure (Fig. 7A–D). Additionally, I examined supporting cell morphology and caspase-3 labeling in the transgenic fish line Supporting-Cell Marker 1 (scm1:GFP), which expresses GFP in all the supporting cells of the lateral line^47^. NM supporting cells appeared intact and their gross morphology comparable to unexposed control immediately following iGluR agonist exposure (Fig. 7E–G). Activated caspase-3 labeling corresponded with hair-cell labeling (Fig. 7F,G), suggesting that activation of downstream cell death pathways in hair cells is not indirectly mediated by supporting-cell damage or death. These data reveal that *ngn1*morphant NM hair cells exposed to KA or NMDA undergo caspase 3-mediated cell death that appears apoptotic.

## Discussion

It has been well established that glutamate excitotoxicity resulting from ischemia or acoustic trauma contributes to cochlear nerve-terminal damage. My observations in the zebrafish lateral-line support the idea that glutamate excitotoxicity also contributes to hair-cell damage and death. The significant findings of this study are i) overactivation of AMPA/Kainate or NMDA-type GluRs leads to hair cell loss, ii) hair-cell loss is independent of afferent and efferent innervation, iii) zebrafish NM hair cells express AMPA/Kainate and NMDA-type GluR subunits and, iv) overactivation of iGluRs contributes to apoptotic hair-cell death.

These observations in the zebrafish lateral line may give insight into the mechanisms of cochlear ischemic injury, which can result from loud noise exposure^1^. Previous studies support that transient cochlear ischemia promotes hair-cell loss^4^, particularly the loss of inner hair cells^48^,^49^,^50^,^51^. It has been proposed that delayed inner-hair cell death following cochlear ischemia is due to excessive glutamate accumulation^51^; glutamate levels in the perilymph increase considerably following ischemic injury^52^, perfusion of the AMPA/Kainate receptor antagonist DNQX protects against inner hair cell loss following transient cochlear ischemia^11^, and treatment of cochlear explants with NMDA receptor blockers protects against hypoxia-induced hair-cell loss^53^. Additionally, inner hair cells have been shown to undergo apoptotic cell death following transient cochlear ischemia^50^, which corresponds to what I observed in iGluR agonist treated zebrafish lateral line. Speculatively, these previous reports combined with the results of this study suggest a model whereby cochlear ischemia and subsequent excess glutamate accumulation may result in over-activation of both AMPA/Kainate- and NMDA-type receptors initiating apoptotic hair-cell death.

The presence of iGluRs in hair cells suggests that activation of autoreceptors on the hair cells themselves initiates intracellular signaling cascades that lead to hair-cell apoptosis. The classical pathway by which glutamate toxicity initiates cell death is by excessive calcium influx leading to mitochondrial dysfunction and the generation of free radicals^54^,^55^. Notably, I observed very little change in [Ca2+]_I_ in dying hair cells exposed to KA (Fig. 6C,D) or NMDA (Fig. 6E,F) and no protective effect from blocking extracellular calcium influx through Ca_V_1.3 channels (Fig. 6G,H), indicating that dysregulation of intracellular calcium is not what drives hair-cell death following excess iGluR activation. There is substantial evidence that specific iGluRs are coupled to the activation of distinct signaling pathways, and it is the triggering of these pathways though excess activation of the receptor rather than an overall elevation in intracellular calcium levels that leads to the activation of programmed cell death^56^,^57^,^58^,^59^. This is further supported by my observations that KA and NMDA have a synergistic effect on hair-cell loss (Fig. 4D), indicating that KA and NMDA initiate hair-cell death through distinct pathways. Additional studies will be needed to verify that hair-cell localized iGluRs conduct calcium current, and to identify the specific downstream signaling pathways they trigger when overactivated.

What might be the physiological role of presynaptic iGluRs in sensory hair cells? Based on previous observations in the central nervous system, presynaptic iGluRs may modulate hair-cell synaptic transmission in response to activity. Presynaptic iGluRs examined in the brain have been shown to generally facilitate synaptic transmission^9^. For example, kainate autoreceptors localized to hippocampal mossy fiber presynaptic active zones facilitate subsequent release of glutamate when activated^60^, and Grin2B containing NMDA autoreceptors in the entorhinal cortex tonically facilitate glutamate release during development^61^. On the other hand, presynaptic iGluRs have also been shown to depress evoked transmitter release^62^; activation of kainate receptors in dorsal root ganglion neurons suppresses their glutamate release^63^. It will be interesting to determine in future studies whether presynaptic iGluRs modulate glutamate release from sensory hair cells and, if so, in what way do they affect synaptic transmission.

In conclusion, sensory hair cells are vulnerable to glutamate excitotoxicity and may be damaged via excess activation of presynaptic iGluRs. The results of this study reveal a potential mechanism of hair-cell death from excitotoxic cochlear injury. Excess glutamate accumulation and excitotoxicity are not unique to ears; glutamate excitotoxicity is responsible for the activation of diverse cell-death pathways in hypoxic-ischemic brain injury as well as a number of neurodegenerative diseases^64^. Determining the mechanisms of excitotoxic pre- and postsynaptic damage in hair-cell organs may provide a better understanding of excitotoxic pathways underlying a variety of neuropathological conditions.

## Methods

### Ethics Statement

This study was performed with the approval of the Massachusetts Eye and Ear Animal Care Committee and in accordance with NIH guidelines for use of zebrafish.

### Fish Strains and Husbandry

Both larval and adult zebrafish were maintained in 14-hour light, 10 hour dark cycle. Adult fish were fed twice daily with GEMMA Micro 300 (Skretting). Embryos for experiments were obtained through paired-mating of Tübingen wild-type fish or the following transgenic lines *neurod:GFP*^65^, -6*myosin6b:ribeyea-mcherry*^66^, -6*myosin6b:gcamp3*^42^, and *scm1:GFP*^47^ and were maintained in E3 embryo media (5 mM NaCl, 0.17 KCl, 0.33 mM CaCl2 and 0.33 mM MgSO4) at 29 °C.

### Statistical analysis

Statistical analysis was performed using Prism 5 (GraphPad Software Inc). The Kolmogorov-Smirnov test was used to test and confirm the normality of data distributions. Statistical significance between multiple conditions was determined by one-way ANOVA and the appropriate post-hoc test; statistical significance between two conditions was determined by unpaired Student’s *t* tests.

### Primary Antibodies

The following commercial antibodies were used in this study: MAGUK (K28/86; 1:500; NeuroMab, UC Davis), acetylated Tubulin (1:1000; Sigma-Aldrich), Parvalbumin (1:2000; Thermo Scientific), HNK-1 (Zn12; 1:500; DSHB, University of Iowa), Otoferlin (HCS-1; 1:500; DSHB, University of Iowa), cleaved Caspase-3 (1:400; Cell Signaling Technology), Gria 4 (1:400; Chemicon); Grik 2 (1:400; Fitzgerald Industries International), Grik 4 (1:400; Genway Biotech Inc.), Grin 1 (1:1000; Synaptic Systems). In addition, the following affinity-purified antibodies generated against *Danio rerio*^26^,^67^ were also used in this study: Ribeye b (mouse IgG2a; 1:2000), Vglut3 (rabbit polyclonal; 1:1000), and Ca_V_1.3a (rabbit polyclonal; 1:1000).

### Whole-Mount Immunohistochemistry and Fluorescence Imaging

5–6 day old larvae were larvae were quickly sedated on ice, then transferred to fixative (4% paraformaldehyde/4% sucrose/0.15 mM CaCl_2_ in phosphate buffer) for either 1 hour (Gria and Grik immunolabel) or 6 hours at 4 °C. Larvae immunolabeled with antibodies for Gria or Grik subunits were permeabilzed in 1% Tween in phosphate buffered saline (PBS) 5 hours at 4 °C, then blocked in PBS buffer containing 1% bovine serum albumin (BSA), 0.5% Fish Skin Gelatin, 2% goat serum, and 0.1% dimethyl sulfoxide (DMSO). All other larvae were permeabilized with ice cold acetone for 5 minutes, then blocked with PBS buffer containing 2% goat serum, 1% BSA, and 1% DMSO. All immunos were incubated with primary antibodies diluted in blocking buffers overnight followed by diluted secondary antibodies coupled to Alexa 488, Alexa 647 (Molecular Probes, Invitrogen), or *DyLight 549* (Jackson ImmunoResearch), and labeled with DAPI (Molecular Probes, Invitrogen). Z-stack images of NMs (spaced by 0.3 μm over 10–12 μm) were acquired with a Leica SP8 confocal microscope using a 63X/1.3 N.A. glycerol immersion objective lens. For each experiment, the microscope parameters were adjusted using the brightest control specimen. Digital images were processed using ImageJ software and Amira 3D software (FEI).

### Morpholino injection

*neurogenin 1* morphant larvae have previously been shown to have the same loss of cranial ganglia as *neurogenin1* mutants. MOs against the start codon of *ngn1* (5′-CCATATCGGAGTATACGATCTCCAT-3′) were obtained from GeneTools (Philomath, OR) and used as previously described^68^. Approximately 1–2 nl of 0.84 mM *ngn1* MO diluted in RNAse-free double distilled water with 3% phenol red was pressure injected into 1-cell stage wild-type and *Tg(neurod:GFP*) outcross embryos. Loss of lateral-line ganglia was confirmed by visualizing the absence of either GFP-labeled neurites in neurod:GFP transgenics or acetylated tubulin immunolabel.

### Pharmacological manipulation of larvae

All of the drugs used in this study were acquired from Abcam. 5–6 days post fertilization (dpf) larvae were exposed to drugs diluted in E3 with 0.1% DMSO for 50 minutes at 29 °C for histology or at room temperature (20–22 °C) during live imaging. E3 with 0.1% DMSO were used as controls. Following drug exposure, larvae were either immediately fixed for immunohistochemistry or allowed to recover for 2 hours or overnight at 29 °C. To determine if there was an additive or synergistic effect between KA and NMDA, the coefficient of drug interaction was calculated per^39^ using the following equation: CDI = AB/(A × B) whereby AB is the ratio of the combined drug group to the control group, and A or B is the ratio of the single drug group to the control group. CDI < 1 indicates synergism, CDI = 1 indicates additivity, and CDI > 1 indicates antagonism. For 30 μM KA + 30 μM NMDA: ((5/11)/((10/11)*(8/11))) = 0.45/0.66 = 0.68 (~0.7).

### FM1-43 Labeling

Free-swimming 5 dpf larvae were immersed in E3 medium containing 3 μM FM 1–43 (n-(3-triethylammoniumpropyl)-4-(4-(dibutylamino)-styryl) pyridinium dibromide; Molecular Probes, Invitrogen)) for 30 s, followed by three rinses in E3 (as described in ref. 32). Larvae were then anesthetized with 0.2 mg/ml of tricaine methanesulfonate (Sigma-Aldrich), placed in a depression slide, and imaged with a Nikon E800 Microscope using 10x/0.45 NA and 40x/0.95 NA dry objective lenses.

### Live-imaging of afferent neurons

5–6 dpf larvae were anesthetized with 0.2 mg/ml of tricaine, then soaked in 1 mg/ml α-bungarotoxin (Alamone labs) for 2 minutes and rinsed in E3. They were kept in E3 prior to imaging until they no longer swam or responded to touch (~20 min.). Larvae were mounted lateral-side on a thin layer of 1–2% low-melt agarose in a 35 mm × 10 mm untreated culture dish to keep them immobilized (Corning) and covered in E3 media. Z-stack images (spaced by 0.6 μm over 5–10 μm) were acquired with a Leica SP2 confocal microscope using a 60x/0.9 N.A. water immersion lens.

### Calcium imaging

5 dpf *ngn1* MO larvae were anesthetized and maintained in 0.2 mg/ml of tricaine or soaked for 5 seconds in 0.6 mg/mL pancuronium bromide (Sigma-Aldrich) and maintained in E3 solution, They were immobilized for imaging in a quick release open bath chamber with a brain-slice harp (Warner Instruments). Methods for imaging and analyzing *myo6b:gcamp3* larvae were based on those previously described using this transgenic line^42^. Images were taken with a Leica SP8 equipped with a HyD hybrid detector at 1400 Hz/6 fps. A 488 Argon laser was used for GCaMP excitation; the laser power was adjusted such that pixel intensities were set at <25% saturation. Baseline readings were taken at 30 sec intervals for 5 min. For drug application, 1/4^th^ of the E3 bath solution was removed and carefully replaced with 4X concentrated drug solution. Additional readings were taken every 30 sec for 50 minutes during drug application. 4-D stacks were converted into maximum intensity projections in ImageJ. Lateral movements (X-Y) of the NMs were corrected using the *StackReg* ImageJ plugin (as described in ref. 69). Following image registration, temporal changes in fluorescence signal were measured within 6 μm diameter ROIs encompassing the basolateral end of selected hair cells. GCaMP fluorescence intensity was calculated relative to the mean baseline intensity of each ROI prior to drug exposure.

### Flow Cytometry Sorting of Hair Cells

Double transgenic 5 dpf larvae (-6*myosin6b:ribeyea-mcherry *+ *neurod:GFP*) were anesthetized with 0.2 mg/ml of tricaine, then transferred to preheated 37 °C TrypLE (1X ; ThermoFisher). Cells were dissociated at 37 °C for 20 minutes with occasional trituration using a glass Pasteur pipette. The cells were spun down in a refrigerated microfuge at low speed (2000 RPM, 3 min.), then resuspended in sorting buffer (1x PBS, 2% Fetal Bovine Serum, 4 mM EDTA, 20 μl/mL DNAse (New England Biolabs), 10 μM Rho/ROCK Pathway Inhibitor (Stem Cell Technologies)). Cell were strained through 35 μm nylon mesh (Falcon/Corning) and sorted at low pressure using a BD FACSAria IIu cell sorter; mCherry- and GFP-postivie cells were detected using 488 nm and 532 nm lasers, respectively. Cells were separated into mCherry-positive, GFP-positive, double-positive, and negative – expressing cell populations, and collected in tubes containing lysis buffer (RNeasy Micro Kit; Qiagen) for subsequent RT-PCR.

### RT-PCR

Total RNA was extracted from isolated cells using the RNeasy Micro Kit (Qiagen). Reverse transcription (RT)-PCR was performed the using RNA to cDNA EcoDry™ Premix Double Primed (Takara). Primers used for each transcript are as follows – **Controls**: *myo6b,* 5′-GGCAAAGCTAGTGGGTGTCT-3′ and 5′-CAGCGCCAGCAAAATGTCTT-3′ ; *syt1a*, 5′-ACTGCCATCATGGGCTATCG-3′ and 5′-ATCCGTCAGCCCTGTTTCTG-3′; β-actin, 5′-AGATGACACAGATCATGTTCGAGACCT-3′ and 5′-GTTGGCATACAGGTCCTTACGGATGTC-3 ; **AMPA receptor subunits**^70^: *gria1a*, 5′-CAGCGCAAACCCTGCGACA-3′ and 5′-CATGGCCAGACCCAGACC-3′; *gria1b*, 5′- TTGAGAGTGCAGAGGATTTGGC-3′ and 5′- CGCCTTCGTCTGTTGTCTTCA-3′; *gria2a*, 5′- TCGAAAGTGCTGAAGAACTGG-3′ and 5′- CGCTCTTCATGTACTGCCACA-3′; *gria2b*, 5′- GAGGACTTGGCAAAGCAAACAG-3′ and 5′- CCACATCTTGTCGAAAAGTGCA-3′; *gria3a*, 5′- ATGGCACCTGGATGACAATGA-3′ and 5′- ACCTCCAACAATACGGCCTGA-3′; *gria3b*, 5′- TGGCAACTGGATGAGACTGATG-3′ and 5′- CCACTATTCTGCCCGATAAGGA-3′; *gria4a*, 5′-GACTTCACACCCAGGTCTCTGTCT-3′ and 5′- CCACATTTTTTCATACACCGCG-3′; *gria4b*, 5′- CCATCGAAAGTGCTGAGGATCT-3′ and 5′-ATTTTCTGACCCGTGCGACTC-3′; **Kainate receptor subunits:** *grik1a*, 5′- GTGCTGTTTGTGATTGCCAG-3′ and 5′- AGATCCTTGACGCATTAGAGC-3′; and 5′- TCAGCACAAAATCTTACCTGAAA-3′ and 5′- GGGAAACAGCTCCACGTAAA-3′; *grik1b*, 5′- CAGCCTGAAAGAGGACGGTT-3′ and 5′- GTCGGTCACGAGTTTGACCT-3′; and 5′- CATCCCGAGGAAAGAGACAA-3′ and 5′- CAGTTCCATCGGCTCAAGAT-3′; *grik2*^71^, 5′- AGCTGATCTTGCAGTGGCGC-3′ and 5′- GGCCGTGTAGGAGGAGATGATG-3′; *grik4,* 5′- CTTACAGCCCTCTGCTTTCG-3′ and 5′- CTGAGCGATAGGGAGTCTGC-3; *grik5,* 5′- AGGGCTGTGACATTAACCCG-3′ and 5′- TGCTCGTTCCCCTCAAACTC-3 and 5′- AGACGCTCTCGTTTGGACAT -3′ and 5′- TCAGACAACACAACCCCAGA -3′; **NMDA receptor subunits:** *grin1a*, 5′- ATTGTGAACATCGGGGCTGT-3′ and 5′-TGAATCGCGTTGGCTTTGTG-3′; *grin1b*, 5′- TGTGGTTCTCCTGGGGTGTA-3′ and 5′-CTCTGCTTCACTGTGGCGTA-3′, *grin2aa*, 5′- GGTGAGAGGAGGACTGTTGC-3′ and 5′-TCATTCACCAGCAGGGTCAC-3′, *grin2ab*, 5′- CTGGAGTGGTGGATGCTCTG-3′ and 5′- TGGCAATGCCGTATCCTGTT-3′; *grin2ba*^72^, 5′- AGAGGGAAGAGTTAGGCGGGATC-3′ and 5′- TGCTCTGTGTCCTGCTCGGGACC-3′; *grin2bb*^72^, 5′- GCAGGATGGGTGGGTTTGGAGG-3′ and 5′- ACTGCTCAAACATCTGGGCGTAGG-3′; *grin2ca*^72^, 5′- AACCACATACCTCACATGCAGCGTTC-3′ and 5′- AGACTGGATACAGGAAGCATGCCATC-3′; *grin3a*, 5′- CGTGGCAGAAACACAACATC-3′ and 5′- TCAGGCAGGATTCCTTCAGT-3′; *grin3b*, 5′- TCGTGTTACTGTGTGTCGGG-3′ and 5′- TTCTGCTCTTTGGCCTCTCG-3′.

## Additional Information

**How to cite this article**: Sheets, L. Excessive activation of ionotropic glutamate receptors induces apoptotic hair-cell death independent of afferent and efferent innervation. *Sci. Rep.* **7**, 41102; doi: 10.1038/srep41102 (2017).

**Publisher's note:** Springer Nature remains neutral with regard to jurisdictional claims in published maps and institutional affiliations.

## Supplementary Material

Supplemental Figures 1 and 2
