# Supplementary material for: Excessive activation of ionotropic glutamate receptors induces apoptotic hair-cell death independent of afferent and efferent innervation
Source: Sci Rep. 2017 Jan 23;7:41102. doi: 10.1038/srep41102 (PMC5255535; doi:10.1038/srep41102)

Excessive activation of ionotropic glutamate receptors induces apoptotic hair-cell death independent of afferent and efferent innervation

Author: Lavinia Sheets

Supplemental Figure 1) A subset of hair cells in KA- and NMDA-exposed NMs form apoptotic bodies prior to cell death.

A-B) Max projection z-stack images from live-imaging of an NM in *neurog1* morphant larvae with hair cells stably expressing GCaMP3. Z-stack images were taken every 30 seconds for 2.5 hours total; 300 μM KA (A) or 300 μM NMDA (B) was applied for 50 minutes, then rinsed with E3 media which remained for the duration of imaging. White dashed outlines indicate dying hair-cells blebbing and fragmenting into apoptotic bodies.

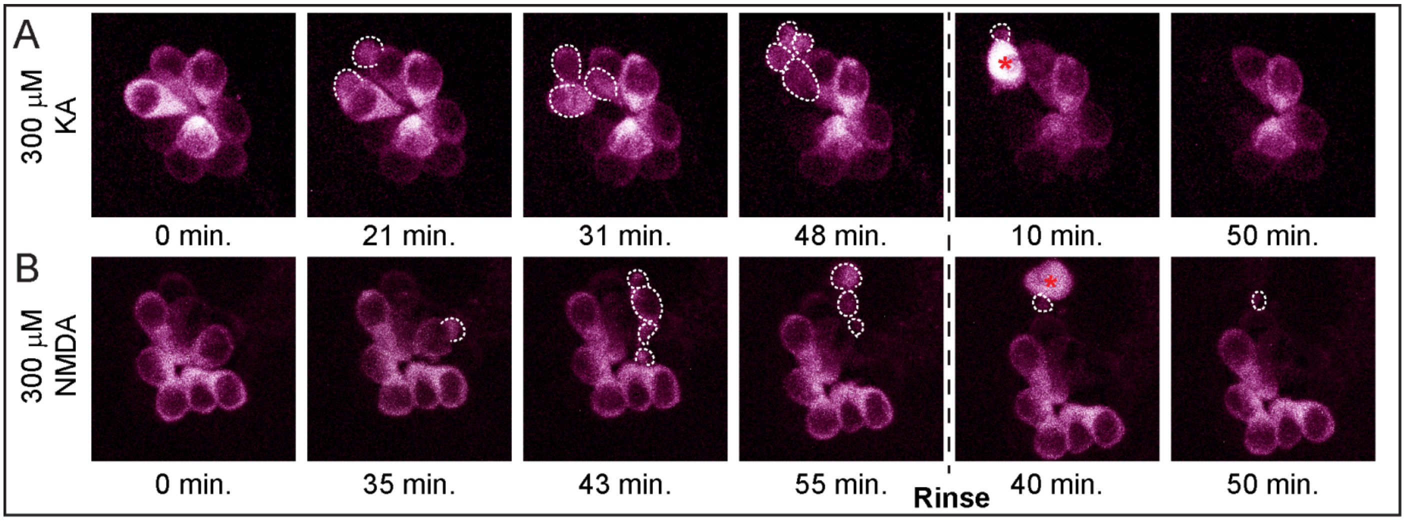

Supplemental Figure 2) Full-length, unedited gels from Fig. 5B.

Top gel: gene expression analysis of isolated mcherry-positive hair cells.

Bottom gel: gene expression analysis of isolated GFP-positive cells (neurons and muscle).

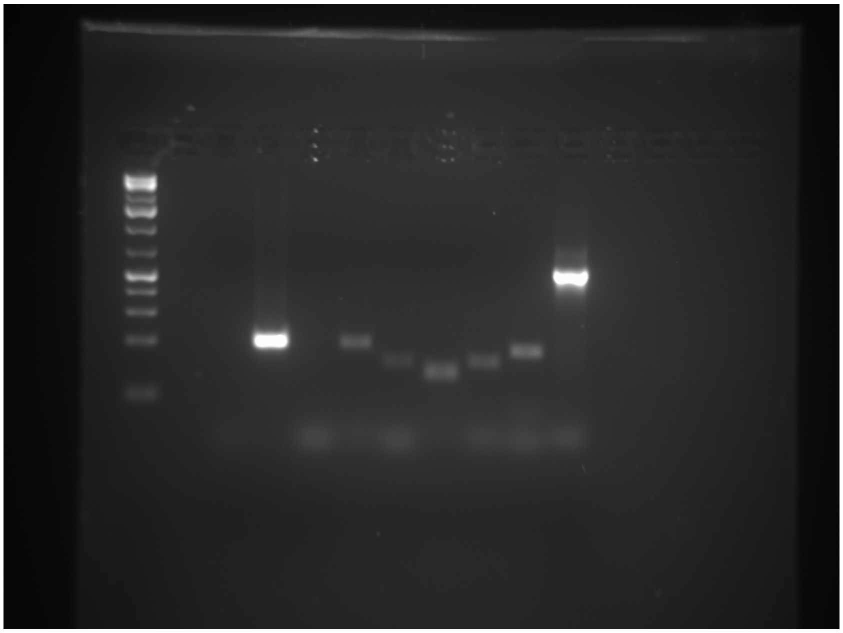

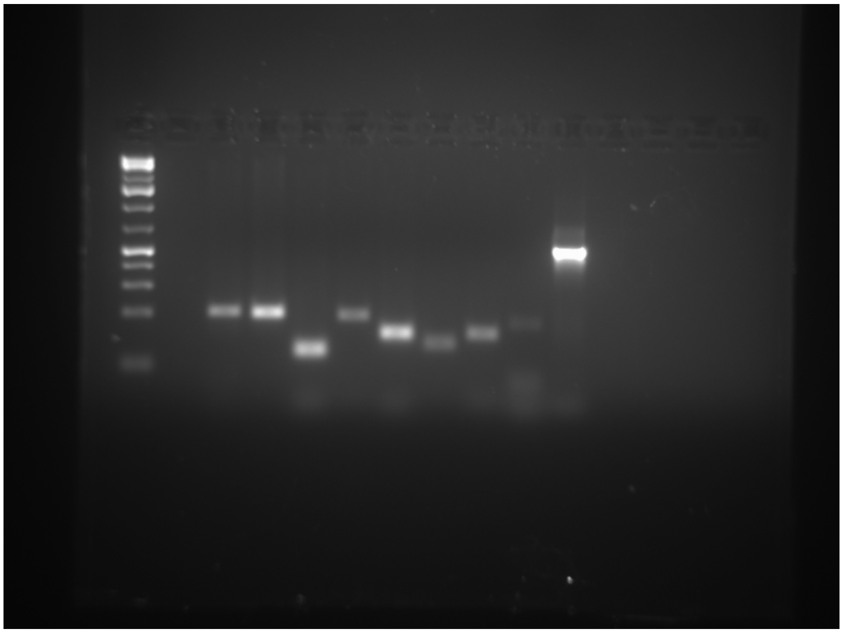

Supplement: Supplemental Figures 1 and 2 [file srep41102-s1.pdf]
